# Evaluating and improving the accuracy of pediatric infusion dose using PDCA combined with HPLC: a quality improvement study from China

**DOI:** 10.1186/s40780-025-00457-y

**Published:** 2025-06-13

**Authors:** Dan Jiang, Min Cui, Baoxia Fang, Fuchao Chen

**Affiliations:** 1 Sinopharm Dongfeng General Hospital, Hubei University of Medicine, Shiyan, Hubei 442008 China; 2 School of Pharmaceutical Sciences, Hubei University of Medicine, Shiyan, Hubei 442000 China

**Keywords:** PDCA cycle, Children, Infusion preparation, High-performance liquid chromatography, Potassium sodium dehydroandrographolide succinate, Quality improvement

## Abstract

**Background:**

Accurate formulation of an intravenous infusion is critical in ensuring its smooth implementation. However, in clinical practice, owing to the diverse reasons for drug preparation, some patients cannot obtain safe and accurate medications, especially in pediatric infusion rooms. Pediatric patients often experience adverse reactions as the dosage administered does not meet the requirements or exceeds the recommended dose.

**Methods:**

Finished product infusion of potassium sodium dehydroandrographolide succinate (PSDS) was used as the study drug. Drug residue samples from the finished product infusion bags were collected randomly in the pediatric infusion room and clinical wards before (from October 2022 to December 2022) and after (from May 2023 to July 2023) the plan-do-check-action (PDCA) cycle intervention. High-performance liquid chromatography (HPLC) was used to determine the drug content. Comparisons of the changes in the proportion of the drug in the infusion were made based on the monitoring results.

**Results:**

After PDCA cycle intervention, the qualified rates of whole, non-whole, and overall infusions increased from 92.95%, 82.68%, and 86.59% to 97.56%, 95.12%, and 96.10% (*P* < 0.05), respectively. The accuracy and uniformity of the infusion preparations significantly improved.

**Conclusions:**

The combination of HPLC and PDCA cycle management can effectively improve the quality of pediatric infusion preparations and enhance their effectiveness.

**Supplementary Information:**

The online version contains supplementary material available at 10.1186/s40780-025-00457-y.

## Background

Intravenous infusion is a critical route of administration in modern drug treatment and plays an irreplaceable role in treating certain diseases and saving the lives of patients [1]. According to literature reports, intravenous fluid administration is the most common invasive procedure extensively practiced in hospital settings [2]. Globally, approximately 25 million people receive intravenous fluid therapy [2]. Compared with adults, children have a lower tolerance for intravenous infusion therapy errors and exhibit more severe physiological responses to these errors. Studies have confirmed that errors in drug dosage calculation may cause adverse reactions in children, and in severe cases, it may even endanger their lives [3, 4]. Most pediatric drugs have standard doses; moreover, regardless of patient size, they are prescribed based on the drug mass per patient weight (e.g., mg/kg) or, in some cases, the drug mass per body surface area (mg/m^2^). This approach is unique to pediatric patients and may partially elucidate why prescription errors are more common in children than in adults [5]. Currently, the supply of clinical drugs for children is limited in China. There are considerable differences in the dosage of drugs for children, and the phenomenon of non-whole infusion dispensing is widespread [6]. Intravenous infusion preparation is an important step in ensuring the quality of infusions. Owing to the noisy environment, complex operation steps, individualized drug dose, large workload, high labor intensity, and other factors during the process of infusion preparation, it is easy to have problems, such as dispensing errors or inaccurate dosage, which poses a great risk to the safety of the infusion [7]. Currently, volumetric method used for monitoring infusion quality by reviewing the volume extracted during allocation to check whether the formulation is precise [8]. Additionally, some studies have demonstrated that intravenous dispensing services by the pharmacy can reduce dispensing errors [9]. Furthermore, gravimetric-based technology [10, 11], converting non-whole drugs into standardized concentrations is used to reduce errors [12, 13]. However, these methods are not commonly used in pediatric wards and do not reflect the quality of pediatric infusion preparations in China. Herein, the use of potassium sodium dehydroandrographolide succinate (PSDS) infusion for injection of commonly used powder formulations in pediatrics was investigated. The quality of this infusion preparation was monitored by high-performance liquid chromatography (HPLC), and the plan-do-check-action (PDCA) method was applied to improve pediatric infusion preparation quality, aiming to provide accurate intravenous infusion for children and ensure the effectiveness.

## Methods

### Chemicals and reagents

Acetonitrile (HPLC grade) was obtained from Tianjin Kemiou Chemical Reagent Co., Ltd., Tianjin, China. Potassium dihydrogen phosphate (purity 99.5%) and phosphoric acid were AR grade (Tianjin Bodi Chemical Reagent Co., Ltd., Tianjin, China). Dehydroandrographolide Succinate reference substances (purity 99.2%) were purchased from the National Institute for Food and Drug Control (Beijing, China). All aqueous solutions including the buffer for the mobile phase were ultrapure water. These were filtered using the 0.45 μm microporous filter membrane.

### Instrumentation and chromatographic conditions

The analysis was conducted using an Agilent 1260, HPLC equipped with a variable wavelength detector. The chromatographic column was Agilent ZORBAX SB-C_18_, 150 × 4.6 mm with 5 μm particle size, supplied by Agilent. The chromatographic data were processed using Open Lab CDS 2. The detection was conducted using acetonitrile-0.02 mol·L^−1^ potassium dihydrogen phosphate (phosphoric acid was used to adjust pH to 3.0) (45:55, v/v) as mobile phase with the flow rate of 1.0 mL·min^−1^ for 15 min. The injection volume and detection wavelength were fixed at 20 μL and 252 nm, respectively. All separations were conducted at 30 °C.

### Study design

The PDCA cycle is divided into four stages: plan, do, check, and action. In the planning stage, we used the PSDS as the research object after a status investigation. The residual liquid left after the completion of the PSDS infusion was collected, its content was detected and qualified by HPLC, and an improvement target was set. In the doing phase, through the establishment of the PDCA cycle group, a series of measures were discussed and formulated to improve the intravenous infusion preparation. In the checking phase, we collected the residues from pediatric infusion after implementing the improvement measures for 4 months for content testing and qualification. The qualification rate after the implementation of the PDCA cycle was used as the observation index. In the acting stage, by comparing the pass rates of the two groups, we explored the impact of the PDCA cycle measures on the accuracy of pediatric infusion content. We optimized the improvements based on the original measures and established a foundation for future PDCA cycle improvements.

### Method of sample collection

All sample preparations were conducted in a sterile environment by nurses in the pediatric transfusion room, pediatric ward 1, and pediatric ward 2, and were supervised by the pharmacist department. During sample extraction, the operators wore sterile gloves and masks to avoid sample contamination. And the products were prepared by Hubei Medical University Affiliated Sinopharm Dongfeng General Hospital.

1. Sample collection before the implementation of the PDCA cycle: From October to December 2022, the residual PSDS solutions in infusion bags of all pediatric patients were collected at 10:30–12:30 a.m. and 5:30–6:00 p.m. Each sample was drawn using a 1 mL disposable syringe and placed in a 1.5 mL EP tube. Label records were made, and the samples were refrigerated at −20 °C until testing.
2. Sample collection after the implementation of the PDCA cycle: From May to July 2023, the residual PSDS solutions in infusion bags of all pediatric patients were collected at 10:30–12:30 a.m. and 5:30–6:00 p.m. Each sample was drawn using a 1 mL disposable syringe and placed in a 1.5 mL EP tube. Label records were made, and the samples were refrigerated at −20 °C until testing.

### Solution preparation

#### Standard solution (2.0 mg∙mL^-1^)

We accurately weighed 50 mg of Dehydroandrographolide Succinate reference substances in a 25 mL volumetric flask, dissolved, and diluted to volume with 5% glucose injection. The solution was then stored in a refrigerator at −20 ℃ until use.

#### Test solution

All samples were diluted according to the following procedure before delivery. We accurately measured 0.5 mL of the sample solution, placed it in a 10 mL volumetric flask, and diluted it to 10 mL using filtered, purified water. Subsequently, 20 μL was drawn for the determination of PSDS content.

### Method validation

This method was validated according to the International Council for Harmonization of Technical Requirements for Pharmaceuticals for Human Use (ICH) and the 2020 edition of the Pharmacopoeia of the People's Republic of China for specificity, linearity, precision, stability, recovery, and robustness [14–16].

#### Linearity

Using the chromatographic conditions mentioned above, series volumes of standard solutions were collected to formulate the series concentrations of 10, 50, 80, 100, 150, and 200 μg∙mL^−1^ and subsequently injected into HPLC to conduct analyses. The linearity of the method was established by triplicate injections in the range of 10–200 μg∙mL^−1^ for PSDS of the nominal analytical concentration. The pediatric dose of PSDS regression equation was defined by plotting the peak area (y) versus concentration (x).

#### Precision

The precision was determined by repeated injection of three different concentrations of high, medium, and low reference solutions of the PSDS (50, 100,150 μg∙mL^−1^) on the same day 6 times, and the results from three straight days were used to determine the inter- and intra-day precisions.

#### Repeatability

The repeatability test was performed by taking appropriate amounts of the PSDS, preparing 6 dilutions with the same concentration (100 μg∙mL^−1^) in parallel, and the peak area was measured using the same detection conditions. The percentage relative standard deviation (RSD) of the PSDS was computed based on the peak area.

#### Stability

Short- (for 8 h at 25 ℃ light exposure condition) and stability of the test and sample solution, and long-term (for 90 days at − 20℃) stability of sample solutions were checked. For the short-term stability test, three different concentrations of high, medium, and low reference solutions of the PSDS (50, 100,150 μg∙mL^−1^) of test and sample solutions (mentioned in Sect. 2.5.2) were collected and placed at 25 ℃ under light exposure conditions. They were sampled at 0, 2, 4, 6, 8, and 12 h. The test solutions were directly determined according to the chromatography conditions mentioned in Sect. 2.2, and the sample solutions were diluted according to the conditions mentioned in Sect. 2.5.2 before determined. For the long-term stability test, three different concentrations of the PSDS (50, 100, 150 μg∙mL^−1^) of sample solution were collected and placed at − 20℃, taken at 0, 7, 15, 30, 60, and 90 d and diluted according to the conditions in section 2.5.2 for measurement. The peak area of each substance and the content of each drug were recorded.

#### Recovery

For the recovery test, various concentrations of the PSDS reference substance solution were spiked into the sample solution. This was determined by the ratio of the difference between the measured and known amounts to the added amount.

#### Robustness

To evaluate the robustness of the detection condition, the influence of three HPLC instruments (Agilent 1260, UltiMate 3000 and SHIMADZU LC − 20A), Chromatographic columns (Agilent ZORBAX SB-C_18_, InertSustain C_18_ column, and Kromasil C_18_), pH values (± 0.2), temperatures (± 1 °C), and flow rates (± 0.02) of the mobile phase on the sample content was investigated.

### Statistical analysis

The relative percentage of PSDS within the range of 90–110% was determined to be qualified. All infusion preparations were enumerated, and the qualified rate of the finished infusion preparations was computed. GraphPad Prism 9.0 was used to draw the scatter plot, which is the relative percentage of infusion drugs, to investigate whether there were differences in the quality of drug preparation in the pediatric ward before and after the implementation of the PDCA cycle. Statistical analysis of the data was performed using SPSS 26.0. The statistical data were expressed as frequency or rate (%). The χ^2^ test was used to compare groups. *P* < 0.05 was considered statistically significant.

### PDCA cycle steps

#### Planning phase

Investigation of the current situation: We assessed the use of pediatric injectable drugs, working environment, and infusion preparation workflow, and subsequently chose the commonly used pediatric infusion of PSDS, which is a representative drug in powder injection, as the study object. Samples were collected according to the requirements described in Sect. 2.4. The PSDS content in the sample was detected using HPLC. The major issues in the preparation process and the content determination results were analyzed, and improvement plans and expected targets were formulated.

**Fig. 1:**
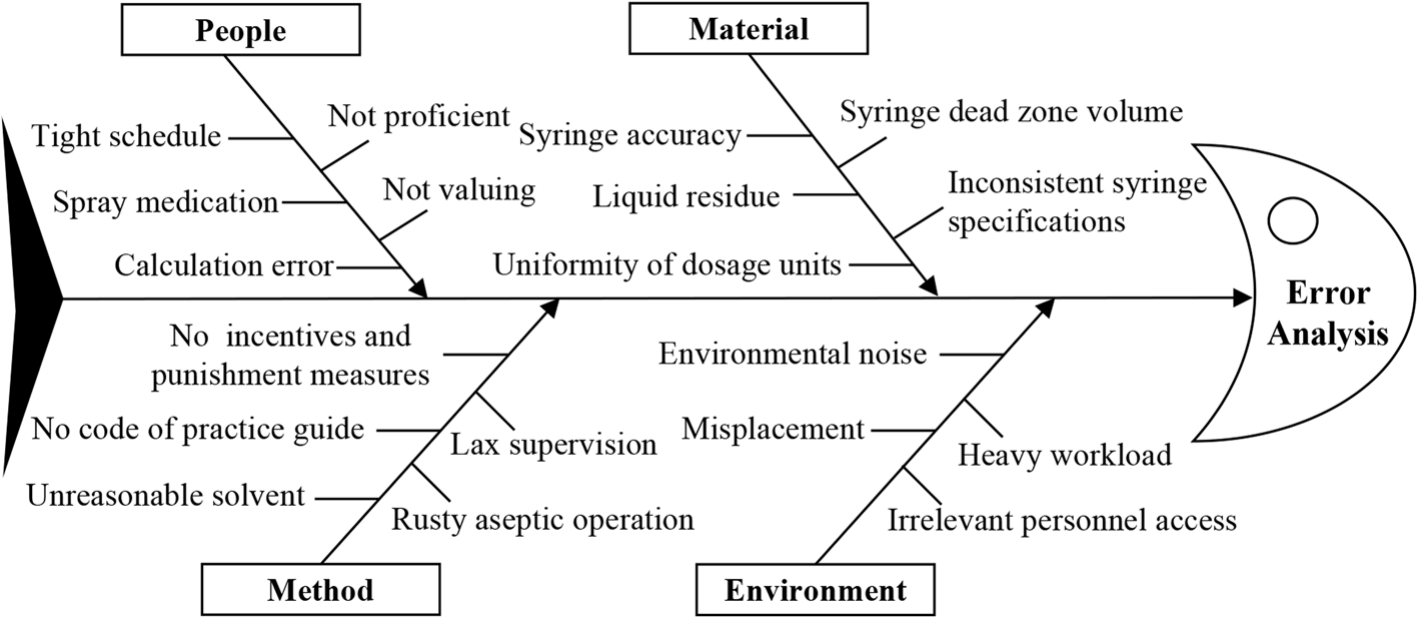
Fishbone diagram of the cause analysis for the low qualification rate in the preparation of product infusion

Cause analysis: According to the monitoring results of the infusion content of finished products for injection in pediatric patients, a root cause analysis of the low qualification rate of infusion preparation was conducted. The diverse factors that may lead to low preparation accuracy are demonstrated using a fishbone diagram (Fig. 1). All factors that may affect the quality of the finished infusion preparation are listed together.

Goal setting: According to the relevant requirements provided by the 2020 edition of the Chinese Pharmacopoeia and the Guidelines for the Construction and Management of Intravenous Drug Dispensing Centers (Trial), this study stipulated that a drug content of 90–110% of the labeled concentration should be used as the qualification standard. Based on the standardized operation procedure, the qualified rate of infusion preparations for pediatric whole and non-whole drug products was increased to > 95%.

#### Doing phase

**Fig. 2:**
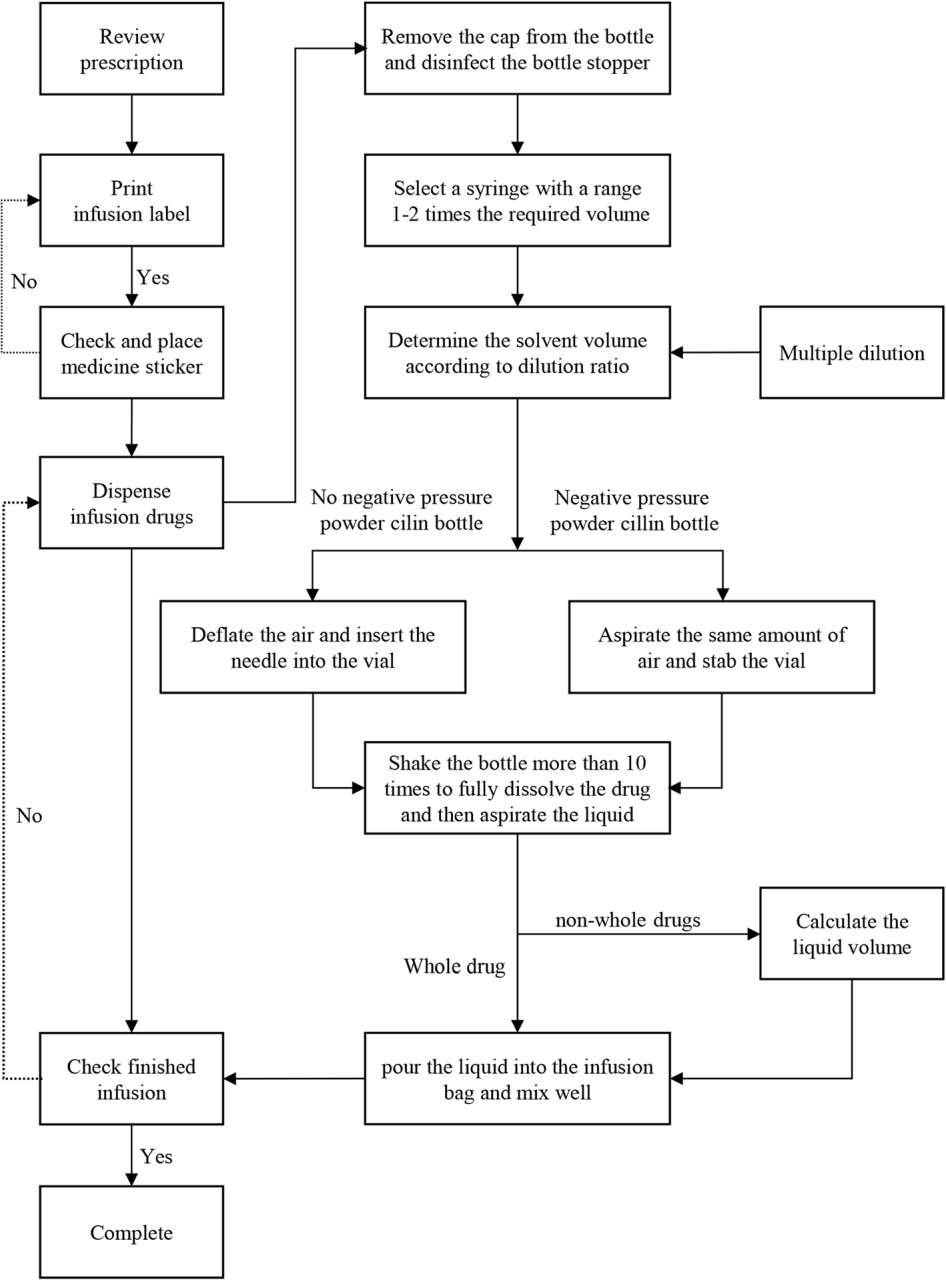
Procedure for the formulation of pediatric infusion drugs

According to the standards and workflow of the centralized intravenous medicine dispensing center in the hospital where the study was conducted. It was combined with the clinical practice of pediatric transfusion dispensing in Chinese hospitals. Through the establishment of the PDCA cycle group, we discussed and formulated measures to improve the quality of intravenous infusion preparation. The following measures were formulated. (1) Surgical procedures for formulating pediatric infusion drugs were formulated, predominantly for selecting syringes and formulating partial drugs. This process is illustrated in Fig. 2. (2) Nursing operations and professional training, such as aseptic operations, risk-related tasks, and infusion preparation process assessment, were strengthened. (3) Modularized the infusion dispensing work area, implemented 6S management, standardized drug placement, took responsibility for people, and maintained an orderly and good working environment. (4) Reasonable arrangement of personnel scheduling, careful implementation of the check management system, and implementation of all links of error registration work were done. (5) Strengthened supervision and inspection, implemented evaluations following relevant provisions, and conducted corresponding rewards and punishments.

#### Checking phase

After 4 months of continuous quality improvement by the PDCA cycle, we collected the prepared samples and computed the qualified infusion formulation rate, then compared the result with that before the PDCA cycle management. Based on the corrective measures and the effect of improving the qualified rate of infusion preparation after practice, we focused on the problems affecting infusion preparation and put forward corresponding measures; then carefully recorded the negligent behaviors in the preparation and held regular meetings to discuss and study the solutions.

#### Acting phase

We summarized the four stages of the PDCA cycle and determined the factors that might affect the quality of infusion preparation. We developed continuous improvement measures and conducted follow-up and effectiveness evaluations to target pervasive and persistent issues to be addressed before the next cycle of quality management.

## Results

### Results of method validation

**Fig. 3:**
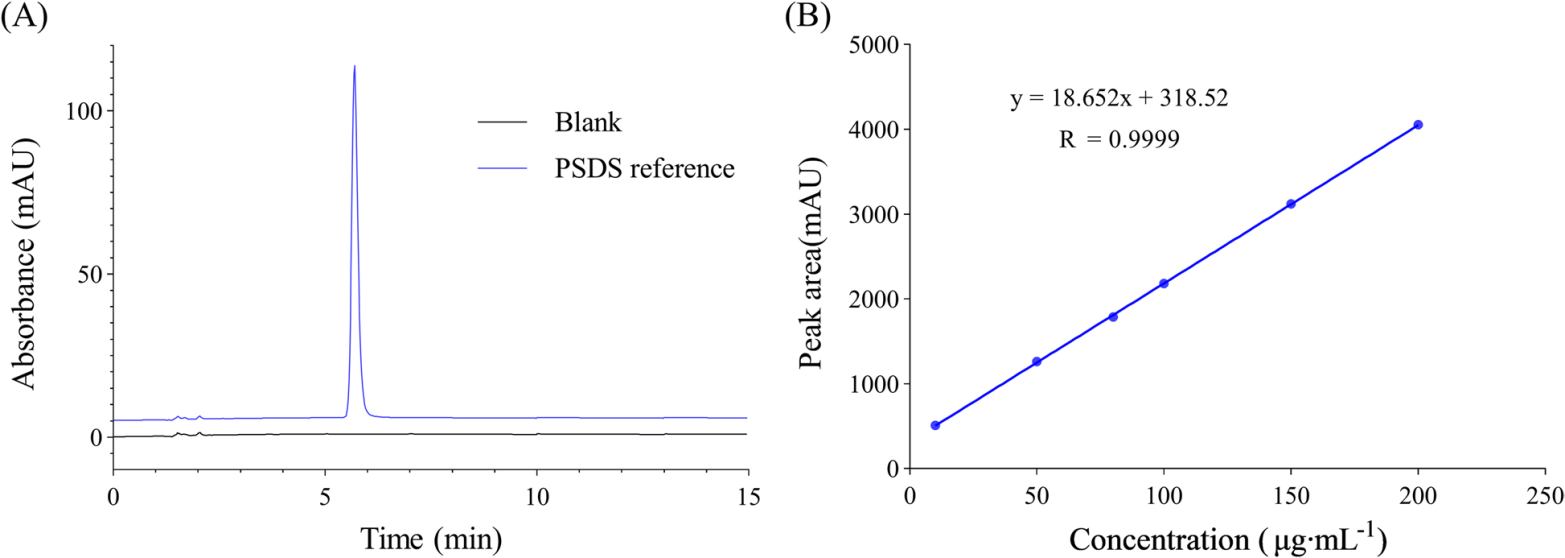
**a** PSDS reference and blank sample chromatogram. **b** The standard curve of the PSDS

The standard solution obtained a good degree of separation in the above chromatographic system, and the blank control demonstrated no interference in the corresponding position. This indicated that the method had good specificity (Fig. 3A). The linear regression equation was expressed as y = 18.652 x + 318.52 with a correlation coefficient of 0.9999 (*n* = 6) (Fig. 3B). The RSD of intra-day and inter-day were 1.2% and 1.7%, indicating good instrument precision, as shown in Table 1. The RSD of the repeatability was determined to be 0.8%. The stability experiment results demonstrated that the test (under conditions of auto sampler) and the sample solutions (ambient temperature) remained stable for 12 h under 25 ℃ light exposure, and the sample solution could be stored at − 20℃ for 90 d. The average recovery rate of the three concentrations was 100.9% with an RSD of 1.5%. This indicated that the method had good accuracy, as detailed in Table 1. In the robustness test, the variation in content under each factor was < 2.0%. This ensured that the HPLC method could be applied for detecting PSDS content.

**Table 1:** The results of method validation

Measured concentrations (μg/mL)	Recovery		Precision RSD	
	Accuracy (%) ^a^	RSD (%)	Interday (%) ^b^	Intraday (%) ^c^
50	102.8	100.4	1.7	1.3
	100.1			
	98.3			
100	101.4	101.0	1.8	1.3
	102.1			
	99.6			
150	101.9	101.2	1.5	1.1
	99.7			
	102.0			

^a^Average of three determinations (every concentration repeated three times)

^b^RSD of nine determinations (repeated three times for three days)

^c^RSD of six determinations (every concentration repeated six times)

### Sample content monitoring results before and after the PDCA cycle

**Fig. 4:**
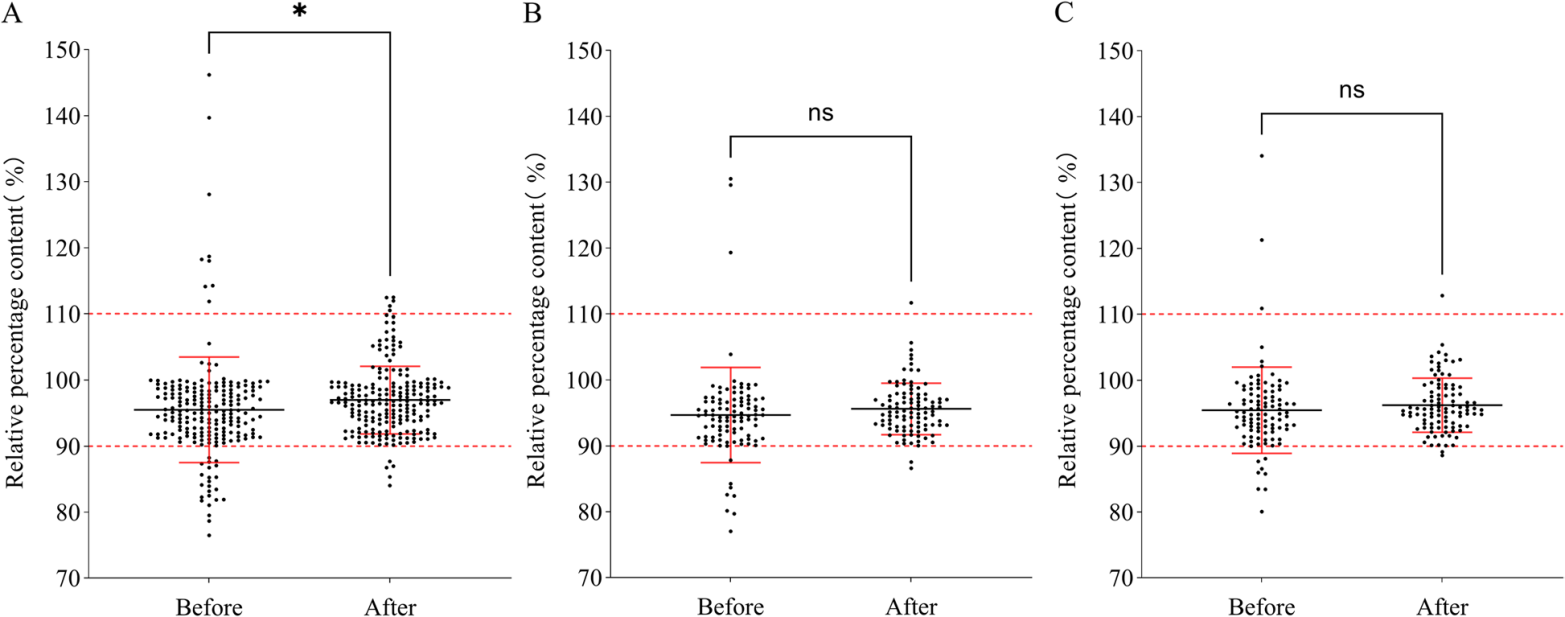
Distribution plot of drug content in PSDS infusion before and after PDCA cycle management. **A** Samples from the pediatric infusion room; **B** Samples from pediatric Ward 1; **C** Samples from pediatric Ward 2

The number of samples selected from the pediatric wards before and after the PDCA cycle and the qualified rate of finished infusion preparation are depicted in Tables 2 and 3, respectively. Scatter charts of the measured sample content distribution in the transfusion room, pediatric ward 1, and pediatric ward 2 are depicted in Fig. 4. The results demonstrated that the qualified rate of the pediatric whole drug was significantly higher than that of the non-whole drug. The qualification rate of the clinical ward was higher than that of the pediatric infusion room. The qualified rate of pediatric infusion was only 86.59% before the PDCA cycle. After the application of the PDCA cycle method to improve the quality of pediatric infusion formulations, the qualified rates of whole, non-whole, and overall infusion formulations increased from 92.95%, 82.68%, and 86.59% to 97.56%, 95.12%, and 96.10%, respectively. The qualified rates of partial and overall infusion formulations increased significantly (*P* = 0.001 and *P* = 0.001, respectively). Additionally, the scatter plot results demonstrated that after PDCA cycle management, the qualified rate of infusion allocation in the pediatric infusion room and clinical ward was significantly higher than that before quality improvement. The uniformity of infusion preparation also improved.

**Table 2:** Sampling of pediatric PSDS finished product infusion

Pediatrics	PDCA	Quantity		
		Whole	Non-whole	Total
Transfusion room	Before	60	150	210
	After	72	138	210
Pediatric Ward 1	Before	49	51	100
	After	48	52	100
Pediatric Ward 2	Before	47	53	100
	After	44	56	100
Totality	Before	156	254	410
	After	164	246	410

**Table 3:** Qualified rate of finished product infusion preparation before and after PDCA cycle management

Pediatrics	PDCA	whole		Non-whole		Total		
		Pass rate	*P*	Pass rate	*P*	Pass rate	*P*	
Transfusion room	Before	91.67%	0.156	82.00%	0.002	82.38%	0.001	
	After	97.22%		94.20%		95.24%		
Pediatric Ward 1	Before	93.38%	0.317	84.31%	0.042	89.00%	0.027	
	After	97.92%		96.15%		97.00%		
Pediatric Ward 2	Before	93.62%	0.339	83.02%	0.020	88.00%	0.016	
	After	97.73%		96.43%		97.00%		
Totality	Before	92.95%	0.051	82.68%	0.001	86.59%	0.001	
	After	97.56%		95.12%		96.10%

## Discussion

Based on previous studies [17, 18], acetonitrile and potassium dihydrogen phosphate were selected to optimize mobile phase composition. The test solution was scanned at the wavelength of 190–400 nm, and the results demonstrated that the maximum absorption wavelength of PSDS was 252 nm. Therefore, the analysis was conducted at a wavelength of 252 nm. A series of concentrations, pH values, ratios of aqueous potassium dihydrogen phosphate solutions, and different chromatographic column types were assessed to ensure good resolution and appropriate retention time of the PSDS. The best result was achieved by comparing the peak shapes and resolutions of the investigated drugs at a pH of 3.0 and acetonitrile − 20 mM KH_2_PO_4_ buffer (45:55; v/v) at a flow rate of 1.0 mL∙min^−1^.

Medical errors in the process of intravenous infusion may harm patients and even lead to death [19]. Children are more sensitive to drugs than adults owing to the imperfect development of their tissues, organs, and metabolic functions. In clinical practice, physicians often administer individualized doses based on the age, weight, and body surface area of a patient. The lack of appropriate dosage forms and specifications for children is a major problem faced by the global medical industry [20]. However, compared to adults, the formulation of partial drugs in pediatric patients is more complicated in terms of dose calculation, less accurate in concentration formulation, and more difficult to validate [21]. Ensuring a more accurate preparation of pediatric finished infusion is critical in ensuring the effectiveness of medication for children. In the past, the methods employed for the preparation of pediatric infusion preparations lacked uniform and standardized clinical criteria. This deficiency rendered it impossible to detect errors in the preparation of some of the infused drugs and did not accurately reflect the true quality of the infusion preparations. Consequently, this study entailed the monitoring of the quality of pediatric infusion preparations through the PDCA cycle, the identification of problems affecting the qualification rate of pediatric infusions, and continuously improving the quality of preparations.

To assess the dispensing accuracy and quality of pediatric infusions, PSDS for injection was selected as the research object. To avoid interference with pediatric clinical infusion treatment, the residual finished infusion samples were extracted from the infusion bag and frozen after the infusion was completed. The time from preparation of all finished infusion products to sampling and storage was 3.64 ± 0.25 h. HPLC was used to detect the drug content. All the samples were tested within 4 h of dilution. The quality of the infusion preparation was analyzed and evaluated by computing the qualified rate. As shown in Table 3, the qualification rate of preparations in the infusion room was lower than that in the wards. We analyzed that the possible reasons were as follows. First, the infusion room had more pediatric patients with diverse disease types, leading to a wider variety of intravenous drugs and greater individual variation in drug dosages. Second, in wards, the types of drugs used for intravenous infusion were relatively fixed, and preparation time was more ample. In Table 3, the qualified rate of pediatric infusion dispensing was 82.68%, which was significantly lower than that of whole drug infusion dispensing. The overall qualified rate of infusion dispensing was also low. We conducted a root cause analysis of the problems and formulated improvement measures, which were mapped using the fishbone method (Fig. 1). The low dose of the infusion formulation may be caused by the lack of drug specifications and dosage forms for pediatric use in China. Additionally, the ages and weights of children differ greatly, making it difficult to determine accurate drug concentrations. During peak hours, the work intensity of dispensing nurses increases, and some nurses simplify the dispensing operation. This can also result in inaccurate infusion preparation. The improper selection of the infusion solvent and syringe may lead to inadequate drug dissolution and inaccurate doses of the aspirated liquid. Based on the issues identified above, members of the PDCA cycle team, which included doctors, nurses, and project researchers, conducted brainstorming and a literature review. They consulted experts to develop improved methods for preparing pediatric infusions. While preparing the whole infusion, the dissolution time was increased, and the direction of the syringe needle was adjusted to completely absorb the liquid in the bottle, thereby avoiding any residue. For non-whole infusion dispensing, the volume of the initial melting solvent was increased, the dissolution time was increased, and syringe specifications were unified to reduce errors. Meanwhile, we used the dilution ratio method to list the dilution ratios of commonly used drugs for direct reference during formulation. After the improvement in PDCA cycle quality, the qualified rates of allocation of whole and non-whole and the overall infusions in the pediatric outpatient infusion room and clinical ward significantly improved; moreover, no infusion allocation errors occurred, and the relative uniformity of the proportion of drugs in the infusion significantly improved.

Internationally, several methods, such as volume review, the gravimetric method [22], and the establishment of the PIVAS have been used to improve the quality of adult infusions [23]. Very few relevant studies that use partial dose drugs have been conducted on special groups such as children. Virginia et al. [24] investigated the accuracy of morphine infusions by detecting the drug concentration of morphine injection, which used volumetric methods for these formulations. It was found that in neonates, there were cases of exceeding the limit in both the ward and the pharmacy-prepared infusion. Lindsey et al. [8] formulated chemotherapy drugs by volumetric method and gravimetric method, and the results showed that the accuracy of drugs formulated by gravimetric method was higher. The gravimetric method refers to the finished product check using the type of the main drug, solvent, and infusion bottle in the finished infusion and adds maintenance quality information to the PIVAS information management system to compute the marked quality of the finished infusion. Using this method, the accuracy of drug formulations can be improved, the error detection rate can be increased, and the occurrence of external errors can be reduced. The previous studies in our research group evaluated the correlation between the gravimetry of infusion, drug residue, and content by testing measures (gravimetric, drug residue, and qualitative and quantitative analysis methods) [25]. The results showed that the qualified rate of aqueous solution drug infusion was significantly higher than that of powder infusion. In the process of preparing powder infusions for infusion, the gravimetric monitoring, residual volume of liquid medicine, or monitoring of drug residue quality can improve the pass rate of infusion preparation and can be used as one of the daily means of monitoring the quality of infusion preparation, but it still cannot accurately reflect the quality of infusion preparation. We suggest that future research should incorporate pediatric infusion preparations into the PIVAS, using gravimetric methods, residual monitoring, or developing real-time monitoring methods. Quality management tools [22, 26], such as quality control circles and PDCA used in hospital management should be utilized to improve the quality of pediatric infusion preparations. We should improve the quality of pediatric infusion preparation, and achieve a normal, sustainable, and low-cost infusion quality monitoring and evaluation method through in-depth research.

This study for the first time used HPLC to detect the content of finished pediatric infusion solutions, conducted a real-world survey, and determined the current status of pediatric infusion preparations. The results of this study verified that this method was effective in improving the quality of pediatric infusion formulations. Simultaneously, combining the PDCA cycle with HPLC can continuously improve the accuracy of pediatric infusion preparation. Intervention measures were implemented before and after infusion preparation using PDCA cycle management. The nursing staff was trained before the preparation, and the drug content was tested and reviewed after preparation. Furthermore, the PDCA cycle team summarized and trained the nurses on the problems that occurred during preparation to improve the quality of subsequent preparations. However, there are certain limitations in clinical application of these results. Although quantitative analysis can accurately analyze the drug content in infusions. Analysis and testing require time and money. It is a post-evaluation method that cannot achieve real-time monitoring and is not suitable for daily evaluation. Despite advancements in domestic and foreign research and the promotion of automatic dispensing robots, automatic dosing robots, and other mixing equipment [27–29], some medical institutions are not equipped with such tools. Currently, Pharmacy Intravenous Admixture Services (PIVAS) are extensively used in China [26, 30].

## Conclusion

The novel method reported here is the first quantitative analysis technology combined with PDCA cycle management, which has effectively improved the quality of pediatric intravenous infusion. The results of this study prove that this method can obtain accurate data on the quality of the infusion and determine its status. The infusion formulation process optimized through the PDCA cycle group can further improve the quality of the infusion formulation.

## Supplementary Information

Supplementary Material 1.

## Data Availability

The data used and/or analyzed during this study are available from the corresponding author on a reasonable request.

## Abbreviations

HPLC: High Performance Liquid Chromatography
PSDS: Potassium Sodium Dehydroandrographolide Succinate
PDCA: Plan-Do-Check-Action
ICH: The International Council for Harmonization of Technical Requirements for Pharmaceuticals for Human Use
RSD: Relative Standard Deviation
