# Supplementary figures and images for: Evaluating and improving the accuracy of pediatric infusion dose using PDCA combined with HPLC: a quality improvement study from China
Source: J Pharm Health Care Sci. 2025 Jun 13;11:49. doi: 10.1186/s40780-025-00457-y (PMC12166566; doi:10.1186/s40780-025-00457-y)

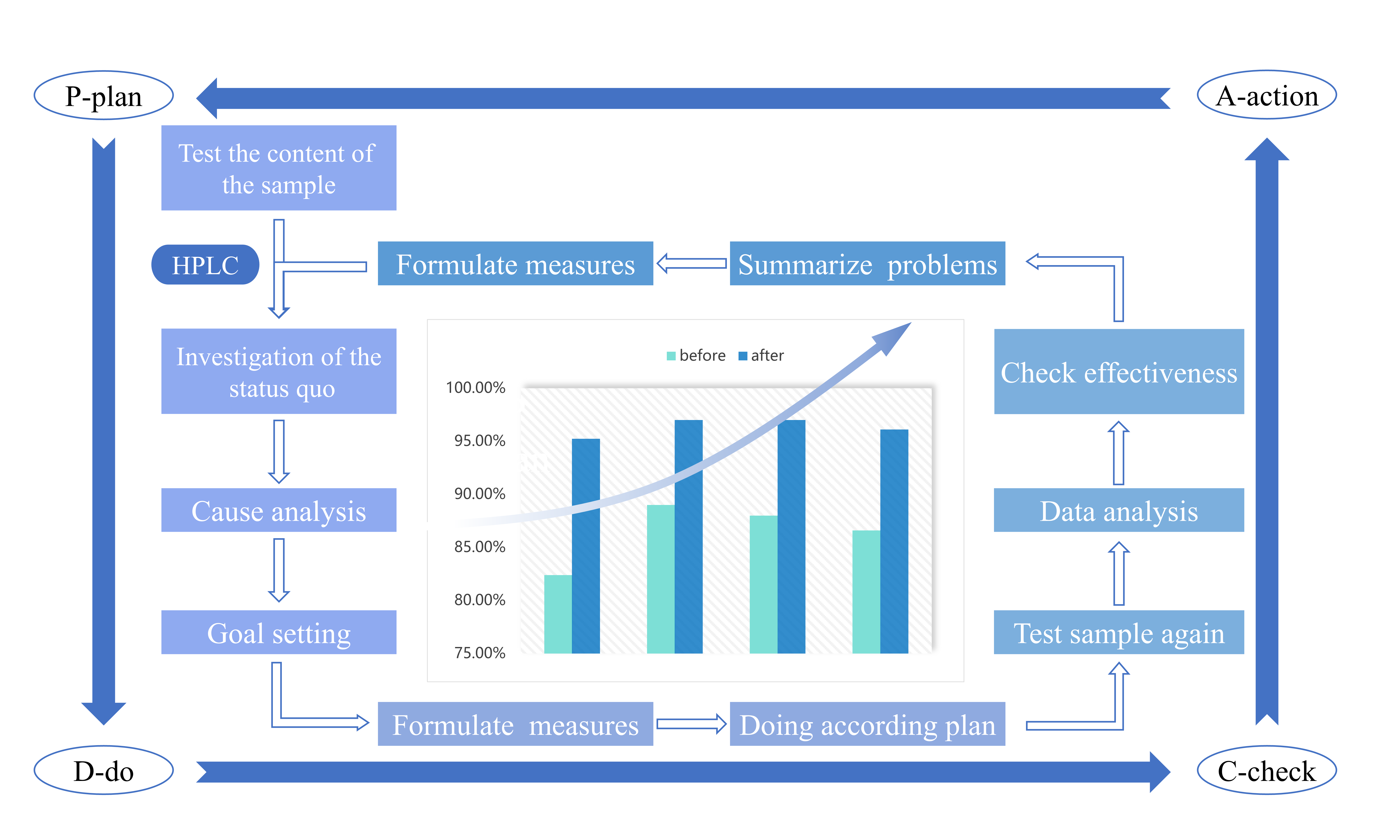

Supplement: Supplementary file 1 — Supplementary Material 1. [file 40780_2025_457_MOESM1_ESM.tif]
